# Heterotopic pancreatic tissue presenting as an unusual cause of gastric outlet obstruction in infancy: a case report

**DOI:** 10.1186/s13256-024-04941-1

**Published:** 2025-04-18

**Authors:** Ahmed Oshiba, Karim Darweesh, Hend Sharafeldin, Mostafa Kotb

**Affiliations:** 1 Pediatric Surgery Department, Faculty of Medicine, Alexandria University, Alexandria, Egypt; 2 Pathology Department, Faculty of Medicine, Alexandria University, Alexandria, Egypt

**Keywords:** Gastric outlet obstruction, Heterotopic pancreatic tissue, Infancy, Case report

## Abstract

**Background:**

Gastric outlet obstruction beyond the neonatal period is an extremely rare condition when other causes such as infantile hypertrophic pyloric stenosis, pyloric atresia, and antral diaphragm are ruled out. Herein, we present the case of a 2-month-old girl who presented with repeated nonbilious vomiting and showed ectopic pancreatic tissue compressing the pylorus of the stomach.

**Case presentation:**

A 2-month-old Caucasian girl who suffered from frequent attacks of projectile vomiting that was not resolved by medication presented to our institute. On ultrasonography, pyloric stenosis was excluded, while on gastrograffin study, there was a hugely dilated stomach with delayed passage of contrast to the distal bowel. On surgical exploration, there was an exophytic pyloric mass compressing the pyloric canal, which was completely excised. Pathological results confirmed the presence of heterotopic pancreatic tissue.

**Conclusion:**

Heterotopic pancreas should always be considered as one of the rare differentials of the causes of gastric outlet obstruction in infants. Although many investigations such as sonogram and upper gastrointestinal series help in diagnosis, histology remains the definitive test in reaching the final diagnosis.

## Introduction

Gastric outlet obstruction (GOO) in infancy and childhood may result from congenital causes, for example, antral diaphragm, pyloric atresia, ectopic pancreatic tissue and infantile hypertrophic pyloric stenosis (IHPS), or acquired causes (peptic ulcer, caustic ingestion, tumor, and chronic granulomatous disease) [1]. With an incidence of up to 1–3.5:1000 per live birth, IHPS is the most frequent cause among them [2]. Whenever IHPS is excluded, the incidence of the latter causes was only 1 in 100,000 live births [3]. IHPS can be diagnosed easily and responds well to Ramstedt’s pyloromyotomy. Other causes of gastric outlet obstruction such as pyloric atresia, prepyloric webs, and diaphragm can be managed by excision of membrane and pyloroplasty. A pyloric mass compressing the pylorus is very rare in infants, but it ought to be considered after ruling out IHPS.

“Ectopic pancreas” is the term used to describe pancreatic tissue located in an organ or tissue that is not anatomically or vascularly connected to the normal pancreas. This condition is also called aberrant pancreas, heterotopic pancreas, or pancreatic rest. The exact mechanism remains controversial, but it has been theorized that it most likely arises congenitally during embryonic development [4, 5].

## Case report

**Fig. 1:**
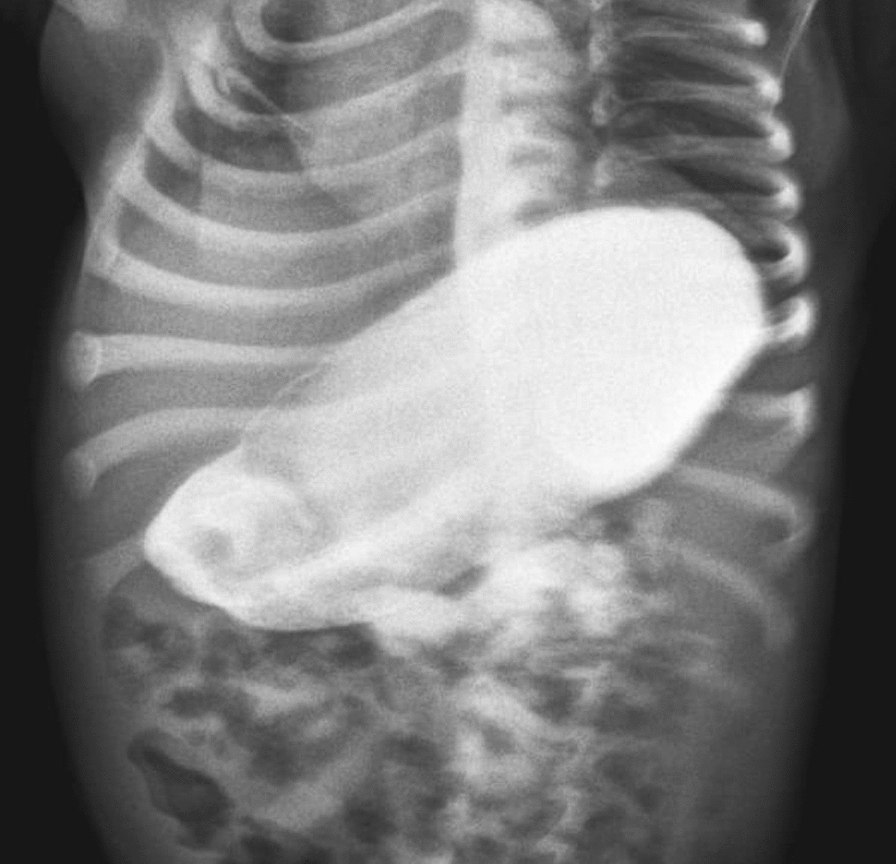
Contrast study showing hugely dilated stomach, prepyloric stricture, and delayed gastric emptying

A 2-month-old Caucasian girl weighing 2.7 kg presented to our institute with continuous projectile nonbilious vomiting for 3 weeks. The frequency of vomiting had gradually increased in the last week. Although the infant was receiving antiemetics, the frequency of vomiting was uncontrollably high, with significant weight loss. Generally, the child was lethargic and severely dehydrated. Abdominal examination revealed fullness in epigastrium with visible peristalsis. Laboratory investigation showed anemia with hypokalemic hyponatremic metabolic alkalosis. Blood urea and serum creatinine were within normal limits. Abdominal sonography showed abnormal thickening in the pyloric canal and gastric dilatation; however, pyloric stenosis could not be excluded. The upper GI contrast study showed a dilated double loop of stomach with significant constriction at prepyloric region and delayed gastric emptying (Fig. 1).

**Fig. 2:**
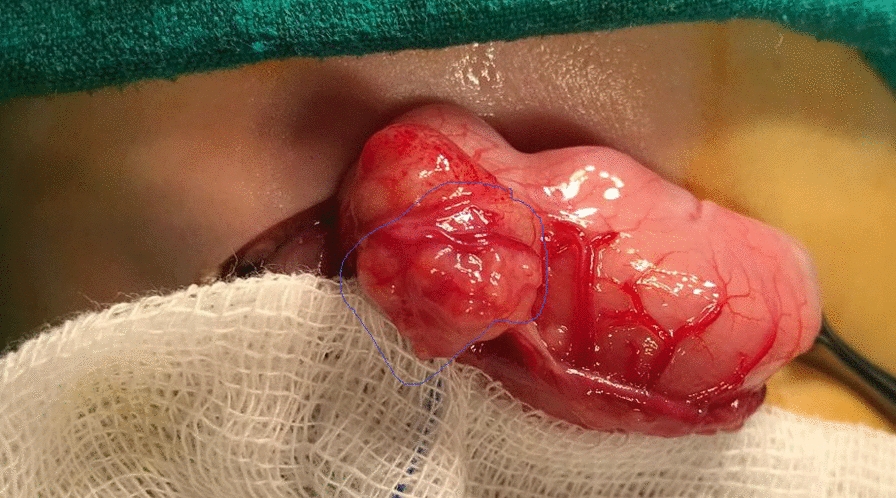
Exophytic pyloric mass, yellowish in color, compressing the pylorus

**Fig. 3:**
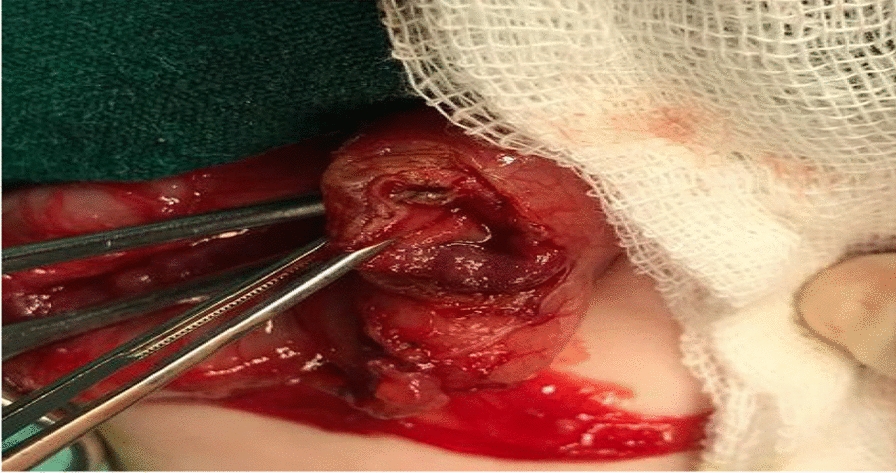
Opened pylorus after complete excision of the mass, closed transversely

**Fig. 4:**
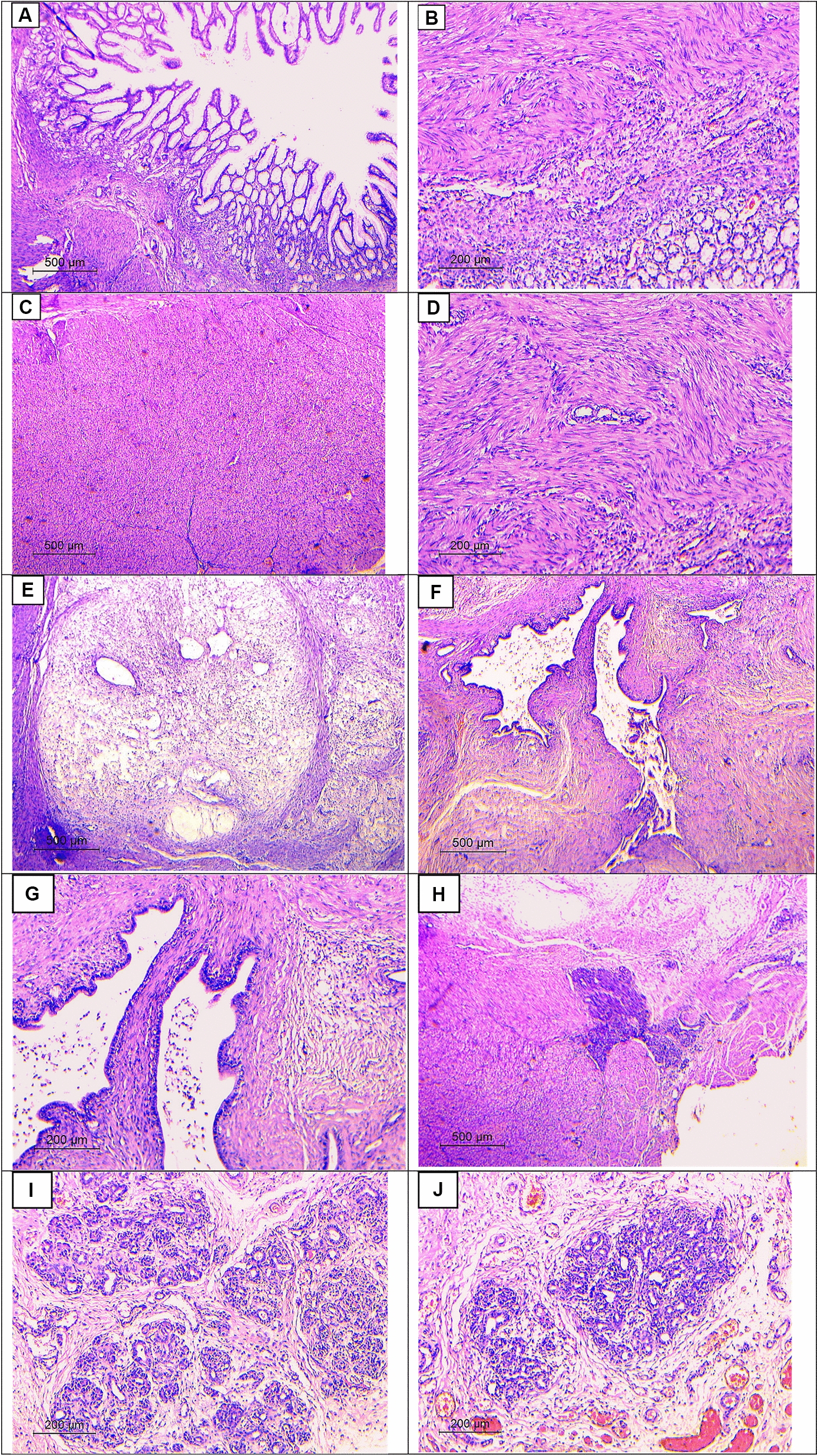
Histopathological examination: (**A**) unremarkable gastric mucosa (hematoxylin and eosin, 40×), **B**) unremarkable gastric mucosa with underlying thickened bundles of muscularis propria (hematoxylin and eosin, 100×), **C**, **D**) thickened bundles of muscularis propria (hematoxylin and eosin, 40× and 100×, respectively), **E**) areas of edema and mild inflammatory infiltrate (hematoxylin and eosin, 40×), **F**, **G**) pancreatic ducts (hematoxylin and eosin, 40× and 100×, respectively), and **H**, **I**, **J**) pancreatic acini (hematoxylin and eosin, 40×, 100×, and 100×, respectively)

The diagnosis of gastric outlet obstruction was confirmed, and the baby was planned for elective laparotomy. Preoperatively, the child was managed with nasogastric aspiration, intravenous fluid, and antibiotics till serum electrolyte correction. On exploration, the stomach was distended with exophytic mass compressing the prepyloric region arising from the external wall (Fig. 2). Longitudinal incision was performed around the mass along the line of pylorus. The mass was firm in consistency, arising from the external wall of the pylorus, and was completely excised. The pylorus was transversally closed (Fig. 3). Patency was checked by passing normal saline through the segment. The postoperative period was uneventful, and the patient started oral feeding on the third day. The pathology of the excised specimen was heterotopic pancreatic tissue causing compression of the pyloric area (Fig. 4).

## Discussion

Gastric outlet obstruction (GOO) in infancy is a relatively rare condition with incidence of 1 in 100,000 live births when IHPS is excluded [1, 3]. The incidence of IHPS is 1.5–3 per 1,000 live births [3]. However, conditions such as gastric web, pyloric atresia, ectopic pancreatic tissue, and duplication of pylorus can also produce the sign symptoms of GOO. The acquired causes of GOO in infants are acid peptic disease, neoplasm, and caustic ingestion, and they are relatively uncommon [2, 6].

There is scant information on the incidence of ectopic pancreas in infancy as a cause of gastric outlet obstruction. In our case, it presented at age of 2 months; however, the lesion is found in 0.2% (1 in 500 surgeries) of abdominal surgeries in adult population, and in a large autopsy studies, the frequency of ectopic pancreas ranged from 0.6% to 13.7%, being commonly seen at 30–50 years of age with male preponderance [7, 8].

Ectopic pancreas is mostly found in the stomach (as in our patient), duodenum, and jejunum; nevertheless, it may also be found anywhere in the digestive tract, intraabdominally, or in the mediastinum or lungs [9]. Involvement of the stomach is seen in 25–40% of cases, most frequently being located along the greater curvature of the gastric antrum [10, 11]. In our case, this abnormal tissue was located in the pyloric region. In the stomach, the submucosal layer is the most common location of the pancreatic rest [12]. Preoperative diagnosis of ectopic pancreas is uncommon, and it usually occurs as an incidental finding [13]. Presentation may, however, be symptomatic in the form of weight loss, epigastric pain (the most common presentation), and vomiting resulting from mechanical gastric outlet obstruction [14]. In our case, the child presented with progressive projectile vomiting and failure to thrive.

Adult patients may also develop complications such as pancreatitis, pseudocyst, insulinoma, and pancreatic carcinoma [8]. The incidence of malignant transformation in ectopic pancreas was reported by Nakao et al. to be as high as 12.7% [15]. Clinically, ectopic pancreas is typically difficult to distinguish from leiomyomas, adenomatous polyps, gastrointestinal stromal tumor (GIST), and even peptic ulcer disease [16]. Endoscopy, sonogram, and computed tomogram may be helpful in diagnosis, but only histology is definitive to differentiate ectopic pancreas from other lesions [17]. In the asymptomatic patient, periodic monitoring is recommended. Patients with symptoms do not respond well to medical treatment; in these cases, localized surgical excision has been demonstrated to be a safe and sufficient procedure, unless malignant transformation has taken place [18, 19]. Prognosis following excision is excellent.

## Conclusion

Ectopic pancreas should always be considered as one of the rare differentials of the causes of gastric outlet obstruction in infants. Although many investigations such as sonogram and upper GI series help in diagnosis, histology remains the definitive test in reaching the final diagnosis.

## Data Availability

The datasets used and/or analyzed during the current study are available from the corresponding author on reasonable request.

## References

[1] Feng J, Gu W, Li M, Yuan J, Weng Y, Wei M, *et al*. Rare causes of gastric outlet obstruction in children. Pediatr Surg Int. 2005;21:635–40. 16041609 10.1007/s00383-005-1472-z

[2] Ormarsson O, Haugen S, Juul I. Gastric outlet obstruction caused by heterotopic pancreas. Eur J Pediatr Surg. 2003;13(06):410–3. 14743331 10.1055/s-2003-44733

[3] Sharma KK, Agrawal P, Toshniwal H. Acquired gastric outlet obstruction during infancy and childhood: a report of five unusual cases. J Pediatr Surg. 1997;32(6):928–30. 9200104 10.1016/s0022-3468(97)90654-0

[4] Burke GW, Binder SC, Barron AM, Dratch PL, Umlas J. Heterotopic pancreas: gastric outlet obstruction secondary to pancreatitis and pancreatic pseudocyst. Am J Gastroenterol. 1989;84:1. 2912031

[5] Pang L-C. Pancreatic heterotopia: a reappraisal and clinicopathologic analysis of 32 cases. South Med J. 1988;81(10):1264–75. 3051429

[6] Ciftci AO, Tanyel FC, Kotiloǧlu E, Hiçsönmez A. Gastric lymphoma causing gastric outlet obstruction. J Pediatr Surg. 1996;31(10):1424–6. 8906678 10.1016/s0022-3468(96)90845-3

[7] Allison J, Johnson J, Barr L, Warner B, Stevenson R. Induction of gastroduodenal prolapse by antral heterotopic pancreas. Pediatr Radiol. 1995;25:50–1. 7761164 10.1007/BF02020846

[8] Mulholland KC, Wallace WD, Epanomeritakis E, Hall SR. Pseudocyst formation in gastric ectopic pancreas. J Pancreas. 2004;5(6):498–501. 15536290

[9] Ormarsson OT, Gudmundsdottir I, Mårvik R. Diagnosis and treatment of gastric heterotopic pancreas. World J Surg. 2006;30:1682–9. 16902740 10.1007/s00268-005-0669-6

[10] Hsia C-Y, Wu C-W, Lui W-Y. Heterotopic pancreas: a difficult diagnosis. J Clin Gastroenterol. 1999;28(2):144–7. 10078823 10.1097/00004836-199903000-00012

[11] Hickman DM, Frey CF, Carson J. Adenocarcinoma arising in gastric heterotopic pancreas. West J Med. 1981;135(1):57. 7257381 PMC1272925

[12] DeBord JR, Majarakis JD, Nyhus LM. An unusual case of heterotopic pancreas of the stomach. Am J Surg. 1981;141(2):269–73. 7457747 10.1016/0002-9610(81)90172-0

[13] Riyaz A, Cohen H. Ectopic pancreas presenting as a submucosal gastric antral tumor that was cystic on EUS. Gastrointest Endosc. 2001;53(6):675–7. 11323606 10.1067/mge.2001.113270

[14] Bromberg SH, Camilo Neto C, Borges AFA, Franco MIF, França LCM, Yamaguchi N. Pancreatic heterotopias: clinicopathological analysis of 18 patients. Rev Col Bras Cir. 2010;37:413–9. 21340256 10.1590/s0100-69912010000600007

[15] Nakao T. Aberrant pancreas in Japan. Med J Osaka Univ. 1980;30:57–63. 7412693

[16] Sukumar N, Teoh C. Heterotopic pancreas in the stomach. Med J Malaysia. 2004;59(4):541–3. 15779591

[17] Armstrong C, King P, Dixon JM, Macleod IB. The clinical significance of heterotopic pancreas in the gastrointestinal tract. Br J Surg. 1981;68(6):384–7. 7237066 10.1002/bjs.1800680606

[18] Ayantunde AA, Pinder E, Heath DI. Symptomatic pyloric pancreatic heterotopia: report of three cases and review of the literature. Med Sci Monit. 2006;12(6):49–52. 16733487

[19] Erkan N, Vardar E, Vardar R. Heterotopic pancreas: report of two cases. J Pancreas. 2007;8(5):588–91. 17873464

